# Effects of an Educational Program for Professional Caregivers on Behavioral Alterations in Nursing Home Residents: Pilot Study

**DOI:** 10.3390/ijerph17238845

**Published:** 2020-11-28

**Authors:** Carolina Pinazo-Clapés, Sacramento Pinazo-Hernandis, Alicia Sales

**Affiliations:** 1 Faculty of Psychology, European University, 46010 Valencia, Spain; carolina.pinazo@universidadeuropea.es; 2 Department of Social Psychology, Faculty of Psychology, University of Valencia, 46010 Valencia, Spain; sacramento.pinazo@uv.es; 3 Department of Developmental Psychology, Faculty of Psychology, University of Valencia, 46010 Valencia, Spain

**Keywords:** dementia, caring professionals, nursing home, behavioural alterations, psychopharmacology

## Abstract

This pilot study aims to analyze the effectiveness of a type of non-pharmacological intervention such as the educating and training of professional caregivers on behavioral alterations and prescription of psychotropic drugs of older adults in nursing homes. One hundred and forty-five people from two nursing homes were randomized to either treatment (educational training program for healthcare professionals) or a no-treatment group. Twenty-two professional caregivers in the experimental group received 20 h of a training program. Five data collection points were collected (pre and post, and three follow-ups, all six months apart). Intervention consisted of the behavioral alterations and psychopharmacological treatment. The analysis of variance for repeated measures showed significant differences in the time-group interaction for the educational program’s effectiveness in reducing behavior alterations and psycho-pharmaceuticals’ record. The results show that an improvement in the educating and training of professional caregivers can reduce behavioral alterations (F3,407 = 9.29, *p* < 0.001, η^2^= 0.063) and prescription of psychotropic drugs (F2,10 = 18.90, *p* < 0.001, η^2^ = 0.117). In addition, these effects are maintained over time. Educating health professionals on ways to care for residents who present behavioral alterations may be one alternative for improving the quality of care that residents receive. Non-pharmacological interventions, besides being individualized and adapted to the needs and experiences of individuals, achieve effects that last longer at low cost. An educational program shows new alternatives to pharmacological intervention, achieving a reduction in behavioral alterations without the costs and effects that psychopharmaceuticals entail.

## 1. Introduction

The recent events caused by COVID-19 have forced us to rethink many aspects that were previously standardized [1]. Specifically, the model of care in nursing homes. The state of alarm imposed in Spain in March 2020 has forced centers to reorganize their protocols, making it even more visible that the biomedical model does not respect the needs, dignity, and freedoms of the elderly. Therefore, it is more necessary than ever to promote actions focused on the model of person-centered care. The important role of care staff in providing practical and emotional support is highlighted [2].

One of the most outstanding aspects of this situation is the treatment and needs of people with neurodegenerative diseases. When talking about neurodegenerative diseases, cognitive changes are the most visible and widely studied part of the illness. However, a set of psychological and/or behavioral symptoms stay in the background. These symptoms have greater repercussions because they pose greater changes and suffering in the person’s psychosocial sphere which significantly affects residents and caregivers [3].

Behavioral and psychological symptoms of dementia (BPSD) refer to a heterogeneous set of psychological reactions, psychiatric symptoms, and abnormal behaviors that occur in people with dementia of any ethology and are considered as relevant as cognitive symptoms because their presence is associated with a poorer quality of life for older people with dementia [4]. BPSD include agitation, aggression, repetitive questioning, sleeping disorders, psychological symptoms such as psychosis, depression, anxiety, and other socially inappropriate behaviors [5].

Around 50% to 90% of people with dementia manifest BPSD [6]. These symptoms lead to a decrease in their autonomy and independence and a worsening of their functionality, which leads to greater load on family members or primary caregivers [7]. In fact, these symptoms also represent a greater burden and a stressful experience, affecting the physical and psychological health of professional and nonprofessional caregivers [8]. This overload leads to increased needs and use of resources or social services and an earlier institutionalization [9]. The BPSD are considered the main reason for caregiver stress syndrome in professionals working in nursing homes [10]. It has also been related to caregiving complications, poor quality care, longer stays, caregiving dissatisfaction, and the stress of professional caregivers [11].

Currently, there are guidelines and associations that recommend choosing non-pharmacological treatments to treat these types of symptoms. But in spite of these guidelines, the bad practice of excess psychotropic use still exists in some care homes [12]. These treatments alter a person’s consciousness, increase the risk of falls and social isolation, and in the case of antipsychotics—due to the sedative effects they cause—can also carry an increased risk of cardiovascular accidents and a higher mortality rate [13]. The effects of pharmacological treatments warn us of the new challenges in the approach to BPSD, more oriented to non-pharmacological therapies.

Dyer et al. [14] highlight the importance of non-pharmacological interventions since they do not entail secondary effects, they imply a lower cost and have been shown to have positive effects. One type of non-pharmacological therapy that is increasingly supported is educational programs for professional caregivers [15,16]. One of the pioneering and highly validated programs is STAR: A Dementia-Specific Training Program for Staff in Assisted Living Residences [17], which continues to be implemented today [18]. STAR programs teach behavioral strategies, dementia education, communication with people with dementia, problem-solving strategies, and implementation of enjoyable events that have a beneficial effect on mood. The authors have produced a version for working with people with dementia living in the community that is the STAR-C and a TELE-STAR version [19], a tele-training project to make the program more cost-effective and accessible to their workers.

The effectiveness of these training programs is now well supported by the literature [20].

Other options or methodologies are the ones that view BPSD as an expression of unmet needs [21], bringing to light the underlying physico-pathological, psychological, and environmental factors. From this line of work, the effectiveness of the program depends on the correct identification of these needs; in other words, in order to achieve the appropriate objective of the intervention, it is necessary to correctly detect the behavioral causes or factors [9]. In short, this BPSD approach, which is based on meeting the needs of each person with dementia, is framed within the model of person-centered care [22,23]. This approach has been shown to be beneficial for older people and also to decrease stress, burnout, and improve job satisfaction for caregivers [24]. Labra et al. [25], argues that interventions focused on the enhancement of caregiving satisfaction would be necessary to formal caregivers without a consanguinity relationship with people with dementia.

It is important to emphasize the importance of the training of caregivers of institutionalized people because what staff do and the way they do it has a very real impact on the overall quality of life of the residents with dementia [26]. Specifically, it is important to train caregivers in the correct management of BPSD following the care model of unmet needs [10] as positive and effective staff–resident interactions optimize the quality of life of people with dementia [18]. In this sense, it is important to emphasize the importance of caregivers ongoing practical training associated with communication skills and care of people with cognitive impairment, which becomes a necessity as well as a priority in the field of dementia in nursing homes [27].

Improving and evaluating training to address BPSD should be a priority approach in the treatment of dementia. Recent studies show that few education and training programs for professional caregivers are well evaluated but it is necessary to objectively measure the improvements offered by these programs and make open reports that allow replication [28]. For this reason, the present work aims to, via a pilot study, evaluate objectively the effects of an educational program for training professional caregivers in the behavioral changes of institutionalized older adults and whether or not the educational program reduces the use and consumption of psychoactive drugs in a group of older adults in nursing homes and based on the model of person-centered care. We will also measure whether this educational program reduces BPSD and if this effect is maintained over time. This is especially interesting since demonstrating that the effects of non-pharmacological therapies are sustained over time and that they decrease the prescription of psychotropic drugs in the long term is an important progression towards reducing the costs of dementia.

## 2. Materials and Methods

### 2.1. Participants

The pilot study’s sample included a total of 145 institutionalized older adults from two nursing homes of the Saleta Care S.L. Group, an organization that leads more than 30 nursing homes in Spain. The educational program was carried out in one of the centers —experimental group, (EG), *n* = 82 and the other center did not experience the program—control group, (CG), *n* = 63. The EG center is a private centre in Spain with a maximum occupancy of 130 places of residence and 40 places of day center. This center has programs that favor person-centered care and has been certified as a center free of physical restraints for more than five years. In parallel, CG was randomly selected in which the educational program had not yet been carried out. The characteristics of the CG center were similar to those of EG. This center also works under the prism of person-centered care and has been free of physical restraints for more than five years.

**Figure 1:**
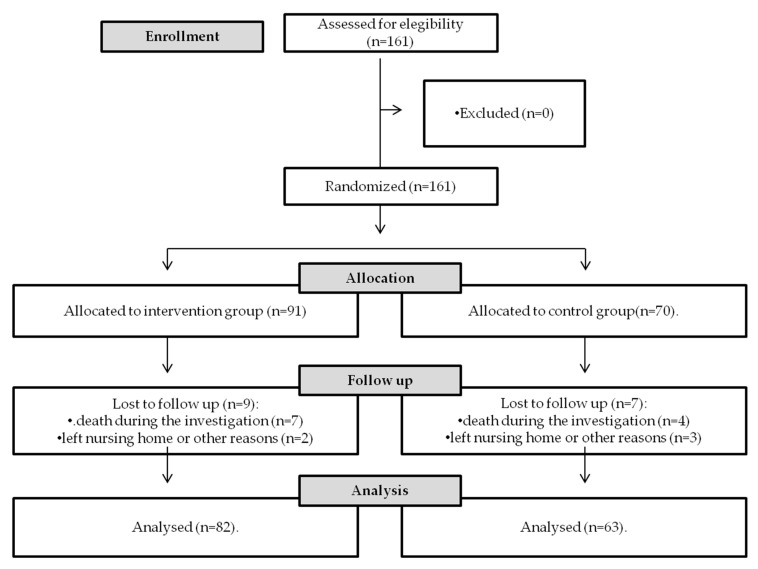
CONSORT diagram.

The inclusion criteria were that the participants of the study had to be already admitted at the time the decision to perform the educational program was done. Participants that left the center for various reasons and could not be monitored during the five evaluations were excluded. Thus, 11 participants were eliminated from our sample because of death during the investigation, and five participants because they left the nursing home or for other reasons. Therefore, in order to evaluate the effects of the program, participants that were to remain at the center for a minimum of two years after the start of the program was a requirement. Figure 1 includes a CONSORT diagram.

The absence of BPSD through the Cumming’s Neuropsychiatric Inventory (NPI) scale as criteria for exclusion was not considered because some patients scored low on the scale as the psychotropic drugs regulated their BPSD. Similarly, not only patients diagnosed with dementia were selected, since BPSDs can present as prodromal symptoms of the disease. In a healthy older adult, it may indicate early dementia or be a risk factor for the future development of dementia [29]. What has been referred to as Mild Behavioral Impairment (MBI) [30].

A quasi-experimental, single-blind design was applied. The nursing homes were randomized to determine where the intervention program would be carried out; the staff could not be blind to the intervention being performed but the patients did not know to which group they belonged. The CG received their usual day-care program; that is, they received the day-care they usually received in the center. Professional participants included 39 caregivers; a total of 22 professional caregivers received the training program (EG) and 19 received their usual day-care program (CG). As an inclusion criterion, participants had to be professionals who completed the program in its entirety and who have been working at the center for at least one year. Furthermore, the professionals who received the training carried out satisfaction and follow-up questionnaires in order to assess their commitment to the implementation of the program as well as to evaluate difficulties.

The educational program was made between time one and time two. After this, the organization provided continuous training for all professionals to ensure the training of all workers during the five times evaluated.

### 2.2. Instruments

Regarding the cognitive level and with the idea of providing a brief and standardized analysis of the mental state, and estimate the existence and severity of cognitive deterioration, the Mini-Mental Status Examination (MMSE) was administered in its Spanish version [31], that consisted of 30 items with a cut-off point of 24. Scores between 0–14 points indicate a severe cognitive impairment, between 15–19 points a moderate cognitive impairment, between 20–24 points a mild cognitive impairment, and between 25–35 points indicate the absence of cognitive impairment.

To assess and evaluate the BPSD, Cumming’s Neuropsychiatric Inventory (NPI) [5] was used. This inventory contemplates twelve symptoms which are delusions, hallucinations, depression, anxiety, apathy, euphoria, agitation or aggressiveness, aberrant motor activity, sleep disorder, eating disorder, and disinhibition. To measure these symptoms, this tool considers the intensity and frequency with which the behavior alterations occur and offers a total score that contemplates the sum of the intensity and the frequency of appearance of all BPSD. There is a version of this test adapted for use in nursing homes, the NPI-NH. This version is a screening tool for nurses or caregivers to assess neuropsychiatric symptoms of residents. In this study, the assessor was an external professional, to ensure the blinding of the assessors. Therefore, the NPI was used.

Psychotropic drugs prescribed at the time of the study were considered and classified into nine categories, as reported by other authors [32,33]: (1) typical neuroleptics; (2) atypical neuroleptics; (3) antidepressants; (4) short-acting benzodiazepines; (5) intermediate-acting benzodiazepines; (6) long-acting benzodiazepines; (7) other hypnotic, sedative or anxiolytic drugs; (8) cholinesterase inhibitors and memantine that we put under the same category; (9) antiepileptics.

The prescription of these psychotropic drugs was accounted for without taking into account the amount of the prescription. For example, it was counted as one if one was taking a short-acting benzodiazepine, counted as two if one was taking two short-acting benzodiazepines, or as three if one was taking two short-acting benzodiazepines and one kind of typical neuroleptic. A blind evaluator carried out the evaluations. The tests were administered in a room at the center and lasted approximately 45 min.

### 2.3. Procedure

All participants in the research, institutionalized older adults from nursing homes or their legal guardians and professional caregivers, signed an informed consent about their participation. Data were analyzed in an anonymous and confidential way.

To perform the study, five time periods were considered (with intervals of six months between each of them). Time 1 (T1) corresponds to the moment prior to the program, T2 was the post measure and the remaining times (T3, T4, and T5) were follow-up measures after the program to analyze the long-term changes.

The objective of the educational program for professional caregivers was to train in the non-pharmacological management of BPSD and to implement protocols to promote the rational use of psychotropic drugs. The design of the educational program featured the main theoretical and practical aspects of institutional intervention for the most prevalent behavioral alterations under the model of unmet needs [14] and aimed to reduce the consumption of drugs.

The 20 h educational program was structured into three weekly sessions of four hours each. In addition, attendees were asked for eight hours of work and readings outside the classroom divided into the three weeks. The contents were composed of the theoretical and practical aspects of the correct guidelines to handle the BPSD for institutionalized older adults. Table 1 summarizes the topics that the program addresses throughout the three sessions.

The educational program was divided into three sessions aimed at the training needs for the management of BPSD and the approaches that have been shown to be most effective according to the one review [34], which are:

— The person-centered approach (session I). Training based on the work of Tom Kitwood [22].
— The behavior-oriented approach (sessions I and II). This approach is based on the importance of the environment and external reinforcements in learning. It is necessary that participants in the course understand that the environment must adapt to each person [35] as well as the relevance of the background and the consequences surrounding each behavior.
— The communication approach (session III). This approach aims to raise awareness about how the way of communicating with people with cognitive impairment, including verbal and non-verbal language, has an influence.
— The emotion-based approach and validation (session III). Training is based on Naomi Feil’s [36] work on validation and the idea of respect and consideration of the feelings of the people with whom one works through individualized attention plans.

In order to facilitate the maximum assistance, the educational program was carried out in the work center and scheduled according to each center’s proposed schedule. The sessions were done in one of the centers rooms with a projector and a laptop by an expert psychologist in the field, and an alien to the study, trained in the person-centered approach.

### 2.4. Data Analysis

In regard to the analyses, t-tests were applied for independent samples and the Chi-square test was used to compare the homogeneity of the groups prior to treatment. In order to analyze the effects of the educational program, an analysis of variance of repeated measures with Bonferroni adjustment was carried out, studying both the simple effects and those of the interaction (group x time). The level of statistical significance considered was *p* < 0.05. The analyses were carried out with the statistical program SPSS 24 (IBM, Amounk, NY, USA).

## 3. Results

### 3.1. Descriptives

In the EG of older adults (*n* = 82), the average age was 87.23 (SD = 6.58) with ages ranging from 70 to 99 years old. CG showed an average of 83.51 (SD = 10.47) with a range of 51 to 101. In relation to gender, EG is composed of 20.7% men and 79.2% women; while CG has 30.2% of men and 69.8% women.

The EG of professional caregivers (*n* = 22) is composed of 13.6% men and 86.4% women. Their ages ranged from 22 to 61 years with an average of 38.27 years old (SD = 12.98). Regarding their jobs, 59.1% of the professionals were nursing assistants, 9.1% technical team professionals, 4.5% nurses, and 27.3% were professionals from other work areas such as administrative staff and maintenance staff. The CG (*n* = 19) is composed of 77.4% women and 22.6% men, from 25 to 60 years with an average age of 39.74 (SD = 11.27). A total of 73.3% of the professionals were nursing assistants, 9.3% technical team professionals, 5.1% nurses, and 12.3% were professionals from other work areas such as administrative staff and maintenance staff.

### 3.2. Tests for Homogeneity

Firstly, tests for homogeneity revealed no significant differences between groups in terms of age—87.39 vs. 83.51; t(142) = 1.544; *p* = 0.668—and gender—χ^2^(1) = 1.69; *p* = 0.193. Significant differences were obtained in regard to cognitive impairment level measured with the MMSE,—t(142) = 2.391, *p* = 0.018—, with an average score of 19.48 (SD = 9.79) in the EG, and with an average score of 15.25 (SD = 11.33) in the CG. Although it is worth mentioning that both scores are within the moderate impairment scale (score between 20 and 11).

### 3.3. Effects of the Program on the Level of BPSD

Once homogeneity was analyzed, the program effects were studied. The intention behind this part of the study, related to BPSD, was to know if BPSD had been reduced after the program and if this reduction was maintained over time.

The analysis of repeated measures showed a significant effect of the program for the time–group interaction (F3,407 = 9.29, *p* < 0.001, η^2^ = 0.063). When comparing the scores of the two groups, the existence of significant differences was verified for the first and last time periods as can be seen in Table 2.

Furthermore, a study of the evolution of the BPSD throughout the different time periods was carried out, analysing the groups with and without the program independently. The analysis showed a significant decrease in BPSD for the EG (F4,135 = 4.45, *p* = 0.002; η^2^ = 0.116) and an increase that was significant for the CG (F4,155 = 3.98, *p* = 0.004, η^2^ = 0.106).

### 3.4. Effects of the Program on the Use of Psychotropic Drugs

From the analysis of repeated measures, we observed a significant effect of the program for the time–group interaction (F2,10 = 18.90, *p* < 0.001, η^2^ = 0.117) regarding the use of psychotropic drugs. In addition, and when comparing the scores of both groups, the existence of significant differences in the last three time periods was verified, as can be seen in Table 3.

Subsequently, a study of the consumption of psychotropic drugs in the different time periods was carried out, analyzing the groups with and without the program independently. The analysis showed a significant decrease of psychotropic drugs for the EC (F5,139 = 2.37, *p* = 0.042, η^2^ = 0.079) and a significant increase for the CG (F5,139 = 8.46, *p* < 0.001, η^2^ = 0.233).

## 4. Discussion

BPSD are common to the disease process causing substantial distress and being the main cause of institutionalization. Professional caregivers in these centers should recognize and manage them in order to alleviate them. For this, the training of caregivers of institutionalized people as an alternative is a good option. This pilot study demonstrated the effectiveness of an educational program to train professional caregivers; the results support the idea that this program decreases BPSD and reduces the use of psychotropic drugs in the institutional setting. These results reinforce the idea that the quality of care has an impact on the frequency and severity of BPSD. In fact, a recent study showed that the presence of BPSD is closely related to the instrumental activities of daily living and, therefore, this association indicates that detecting BPSD and changing lifestyle and care style can promote the autonomy and independence of older people [37].

As indicated by Spector in a systematic review [34], staff training can reduce BPSD and improve staff behavior. However, no links were found between the theoretical orientation of training and outcome. The authors conclude that there are training programs centered on one of the theoretical orientations but there are few programs that unite several approaches. The present study highlights that a combination of them has been worked on: the behavior-centered approach, its background, and consequences, the approach focused on verbal and non-verbal communication used by people around them, and the person-centered approach and the emotion-centered approach. On the other hand, most of the studies are based on self-reported improvement or some studies did not include a control group [16]. Finally, other highlights of this study are the five follow-up measures. The occurrence of BPSD was significantly different between GC and EG for time 1 and time 5. This change in BPSD is the result of an educational intervention and a key finding for this study. Not measuring these five times has been considered a limitation in other studies [15]; in the case of our study, these measures show the effectiveness of the intervention to improve caregiver competence and knowledge through intensive training and demonstrate that the effects of this training are sustained over time.

In fact, the different measurements made at different time periods that demonstrate that this change is maintained over time, supports some studies [25] who measured the impact of an intervention in quality of care. The results were positive and were maintained for six months after the intervention. Some authors evaluated the effectiveness of a training program to alleviate the agitation of residents, noting that not only was training effective but also, the decrease in aggressiveness was maintained over time [38]. Monitoring the results after the intervention in order to evaluate the continuation of the effect is important, given that authors [39] state that the effect of training caregivers lasts longer after the intervention than the effect of psychotropic drugs that must continue to be administered to maintain their results. All this supports once again the need to design non-pharmacological interventions.

The results of this study indicated a significant decrease in BPSD for the EG and a significant increase for the CG. Similar results to these have been found in other studies [40]. There are data in the literature that support that age is positively related to increased cognitive impairment and a greater cognitive deterioration is related to a greater presence of BPSD [41], which can explain this significant increase in the control group.

In addition, the findings suggest that this type of intervention also reduces the use of psychotropic drugs in the institutional setting, which supports the findings in others works [33], that demonstrated that with adequate training, caregivers could reduce depression and anxiety symptoms. Several reports have indicated that the use of antidepressants and benzodiazepines is very high for the treatment of these symptoms [42]. The risks associated with the excessive use of psychotropic drugs in older adults are especially relevant. These practices can increase the risk of suffering cardiovascular accidents or increase the degree of dependence [43]. In fact, polypharmacy has been shown to be related to an increased risk of falls [44], so a decrease in the use of psychoactive drugs could be related to a lower registry of falls in the center, which in turn could be related to a better functional status. This aspect is especially relevant when we work with people who live in nursing homes and where falls are a very frequent problem.

In conclusion, it seems clear that there is a need for a change in nursing home care models. A shift towards a person-centered care model that takes into account the needs and desires of each individual that will improve cognitive and functional aspects and directly affect their quality of life [45]. In 2017, the World Health Organization proposed a challenge to which they entitled “medication without harm” [46] with the aim of bringing together expert opinions, protocols, and consensus to guide professionals in their professional practice. These guidelines, as well as the CHROME criteria [12], advise the design of more non-pharmacological interventions like the one proposed in this work as a first line of intervention when acting in the presence of BPSD.

Studies on the use of psychotropic drugs in institutionalized older adults [47] found that between 37% and 78% of people had at least one prescribed psychopharmaceutical. Between 21% and 54% of people took neuroleptics, between 15% and 31% took sedatives, between 9% and 38% took anxiolytics and between 19% and 47% took antidepressants daily. In fact, the presence of BPSD could, in many cases, lead caregivers to be the ones to most request the use of physical and chemical restraints in the center. Therefore, having knowledge of improvements in the environment (lighting, space design) or the adverse effects of the abusive use of psychotropic drugs can lead to better management and better treatment of people with behavioral disorders, and in doing so, avoid transferring the need to evaluate the prescription of psychotropic drugs to the medical team.

This idea is strengthened with the other results that found that the more knowledge healthcare professionals had about restraints, the more negative their attitude towards them was and the more likely they were to reject them [43]. This is especially important and is consistent with a recent study that found that the increase in healthcare professionals’ knowledge about restraints in the hospital environment, significantly decreases the use of them [48]. Therefore, the training aimed at professionals understanding and knowing the risks posed by restraints is fully recommended to encourage the rational use of psychotropic drugs [40].

This type of intervention depends a lot on organizational factors such as the care model of the centers, the organizational model, or the business culture [33]. Its implementation is sometimes complicated so its positive effects, as shown in this work, should be highlighted so that the effort is made to get them in motion.

The results have shown that significant effects on behavior and use of psychoactive drugs have been achieved. This aspect is relevant considering the high costs involved in dementia care [45] and that this type of intervention can significantly reduce direct and indirect costs.

As limitations and suggestions for future studies, it is important to highlight the size and origin of the sample. It could be interesting to have a greater number of centers of different kinds (public and/or private) from different geographical areas in order to increase and generalize the results. The study should be carried out in multiple nursing homes using a cluster randomized trial design. Another important limitation is the availability of a more reliable quantitative measure of drug use. These aspects of the design must be improved in future studies in order to generalize the results of this pilot study.

On the other hand, it is important to highlight that the appearance of BPSD and the prescription of psychotropic drugs could be related to a greater cognitive and functional impairment [49]. Therefore, in future works, it would be interesting to study if staff training could positively influence this aspect by favoring a more rational use of psychotropic drugs.

In addition, it would be interesting to include the approach to the emotional sphere, functional status, level of dependence, and physical changes in future interventions. In this sense, it would also have been interesting to add content to the training program that would contemplate the importance of structured, pleasant activities and events, or more social support as already introduced by the STAR project [50]. Finally, it would have been interesting to be able to evaluate the training in professional caregivers with a follow-up measure after the completion of the program to evaluate the effectiveness of this training and staff engagement with the intervention.

## 5. Conclusions

This study has been shown to reduce the prescription of psychotropic drugs in older people living in nursing homes. The use of these treatments is increasing and produces very harmful side effects for older adults. The training and education program for professionals evaluated in this study manages to reduce the appearance of behavioral problems associated with dementia and also reduces the consumption of psychotropic drugs. Moreover, these effects are maintained over time, so that the application of this program can have an impact on the cognitive and functional aspects associated with dementia, and can also help to reduce the costs of this disease and will promote a model of care based on the person-centered approach. This model is very necessary in this situation caused by COVID-19 in nursing homes.

## Figures and Tables

**Table 1:** Educational program contents.

Sessions	Contents
**Session I**	Aging, dementia, and cognitive impairment
	Behavioral and psychological symptoms of dementia and biopsychosocial repercussions
	Person-centered care in residential caring
	Biopsychosocial model and importance of the environment
	Introduction to the risks of physical and chemical restraints
**Session II**	Conceptualization of each behavioral and psychological symptoms of dementia
	Possible precipitants
	Management and case studies
**Session III**	Importance of communication
	Guidelines to facilitate verbal communication
	Non-verbal communication
	Aspects to avoid when dealing with people with dementia
	Infantilization, ageism, and ostracism
	Validation method and its benefits
	Conclusions

**Table 2:** Means and standard deviations of groups on the level of BPSD and univariate statistics comparison.

Time	Experimental Group Mean (SD)	Control Group Mean (SD)	F *	*p*-Value	η^2^ **
T1	10.13 (11.67)	5.18 (5.99)	9.01	0.003	0.061
T2	9.68 (9.99)	7.65 (7.93)	1.69	0.195	0.012
T3	9.81 (11.27)	7.28 (7.99)	2.19	0.141	0.016
T4	9.10 (9.82)	8.08 (8.93)	0.39	0.530	0.003
T5	6.36 (7.45)	10.10 (10.37)	5.90	0.016	0.041

*: Fisher Value; **: Eta-squared.

**Table 3:** Means and standard deviations of groups on psychotropic drugs and univariate statistics comparison.

Time	Experimental Group Mean (SD)	Control Group Mean (SD)	F *	*p*-Value	η^2^ **
T1	1.65 (1.30)	1.60 (1.26)	0.06	0.798	0.000
T2	1.52(1.21)	1.85 (1.57)	2.05	0.154	0.014
T3	1.47 (1.19)	2.01 (1.57)	5.51	0.020	0.037
T4	1.43 (1.16)	2.07 (1.60)	7.71	0.006	0.051
T5	1.35 (1.22)	2.27 (1.65)	14.68	0.000	0.093

*: Fisher Value; **: Eta-squared.
